# Explanation of efficient quenching of molecular ion vibrational motion by ultracold atoms

**DOI:** 10.1038/ncomms11234

**Published:** 2016-04-18

**Authors:** Thierry Stoecklin, Philippe Halvick, Mohamed Achref Gannouni, Majdi Hochlaf, Svetlana Kotochigova, Eric R. Hudson

**Affiliations:** 1 Université de Bordeaux, Institut des Sciences Moléculaires, UMR 5255 CNRS, 33405 Talence, France; 2 Université Paris-Est, Laboratoire Modélisation et Simulation Multi Echelle, MSME UMR 8208 CNRS, 5 bd Descartes, 77454 Marne-la-Vallée, France; 3 Department of Physics, Temple University, 1925 N 12th Street, Philadelphia, Pennsylvania 19122, USA; 4 Department of Physics and Astronomy, University of California, 475 Portola Plaza, Los Angeles, California 90095, USA

## Abstract

Buffer gas cooling of molecules to cold and ultracold temperatures is a promising technique for realizing a host of scientific and technological opportunities. Unfortunately, experiments using cryogenic buffer gases have found that although the molecular motion and rotation are quickly cooled, the molecular vibration relaxes at impractically long timescales. Here, we theoretically explain the recently observed exception to this rule: efficient vibrational cooling of BaCl^+^ by a laser-cooled Ca buffer gas. We perform intense close-coupling calculations that agree with the experimental result, and use both quantum defect theory and a statistical capture model to provide an intuitive understanding of the system. This result establishes that, in contrast to the commonly held opinion, there exists a large class of systems that exhibit efficient vibrational cooling and therefore supports a new route to realize the long-sought opportunities offered by molecular structure.

## Abstract (web summary)

To exploit the internal structure of molecules it is necessary to reach very low temperatures, but this is made impractical by long relaxation times for molecular vibrations. Here the authors provide a theoretical description for the existence of a large class of systems exhibiting efficient vibrational cooling.

## Graphical abstract

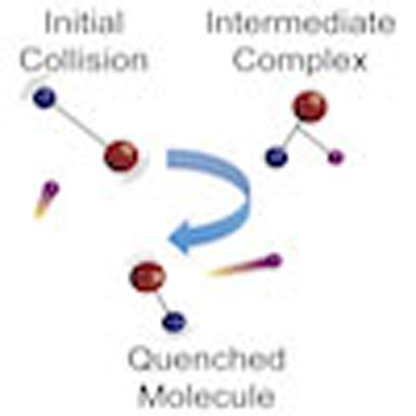

The internal structure of molecules offers a host of scientific and technological opportunities^1^, including the manipulation of quantum information, critical insight into quantum chemistry and improved tests of the Standard Model. To utilize this potential of molecules typically requires the preparation of molecular samples at very low temperatures, where only a single quantum state is occupied. Unfortunately, experiments attempting to reach these temperatures by buffer gas cooling have found that although the molecular motion and rotation are quickly cooled to the cryogenic temperature^2^,^3^, the molecular vibration relaxes at impractically long timescales^4^. However, in a recent study^5^, sympathetic cooling through collisional interaction with *laser-cooled atoms* was demonstrated to be an alternative and efficient approach to quench molecular ion vibrational motion. Although this result was predicted based on a semi-classical argument^6^, it had not been verified by detailed quantum mechanical calculations. Further, it was not known how widely this technique could be applied, nor how to estimate its efficiency for other systems.

Here, we perform a detailed theoretical study of the Ca-BaCl^+^ system and compare to the recent experimental results, as well determine efficiency criteria to predict vibrational quenching rates in similar systems. Specifically, we first build an analytical model of the potential energy surface (PES) of the Ca- BaCl^+^ collision using a large grid of *ab initio* points, taking special care to accurately describe its long-range behaviour, which has an important role at very low collision energy. We then perform close-coupling calculations of the vibrational quenching of BaCl^+^ by collisions with Ca atoms. Although such calculations are nowadays straightforward and relatively fast, it is highly computationally demanding in the case of Ca-BaCl^+^ owing to the large mass and bonding energy. We therefore give a brief account of the method used to make the calculations feasible in a reasonable amount of computer time and compare the results with experiment. In addition, we use a scattering model based on quantum defect theory (QDT) with generalized short-range boundary conditions to gain an insight into the vibrational cooling of the molecular ions by the ultracold atoms. Finally, we compare the close-coupling vibrational quenching results with those obtained for four other similar systems: He-N_2_^+^, He-NO^+^, He-CH^+^ and Ar-NO^+^ and propose a very simple statistical capture model, which reproduces the close-coupling results and provides a simple means to estimate the efficiency of vibrational quenching for a given system. This result establishes that, in contrast to the commonly held opinion, there exists a large class of systems that exhibit efficient vibrational cooling and therefore supports a new route to realize the long-sought opportunities offered by molecular structure.

## Results

### Ca-BaCl^+^ PES calculation

To calculate the Ca-BaCl^+^ vibrational quenching rate, we first built an analytical model of the PES of the Ca-BaCl^+^ collision using a large grid of *ab initio* points. The electronic ground state of the CaBaCl^+^ complex, a ^1^A′ state, was calculated with the MOLPRO programme suite^7^ (see the Methods for details) on a three-dimensional (3D) grid of points in the Jacobi coordinates space *r*, *R* and *θ*. Here, *r* represents the BaCl^+^ bond length, *R* the distance between Ca and the centre of mass of BaCl^+^, and *θ* the angle between **r** and **R**, with the linear structure Ba-Cl-Ca corresponding to *θ*=0°. The functional form *V*(*r,R,θ*) of this PES is defined as the sum of the interaction energy *V*_I_ between Ca and BaCl^+^ and the potential of the isolated diatomic BaCl^+^, *V*_BaCl_:


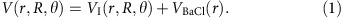


These terms are found by interpolation over the *ab initio* calculations (see the Methods for details).

**Figure 1:**
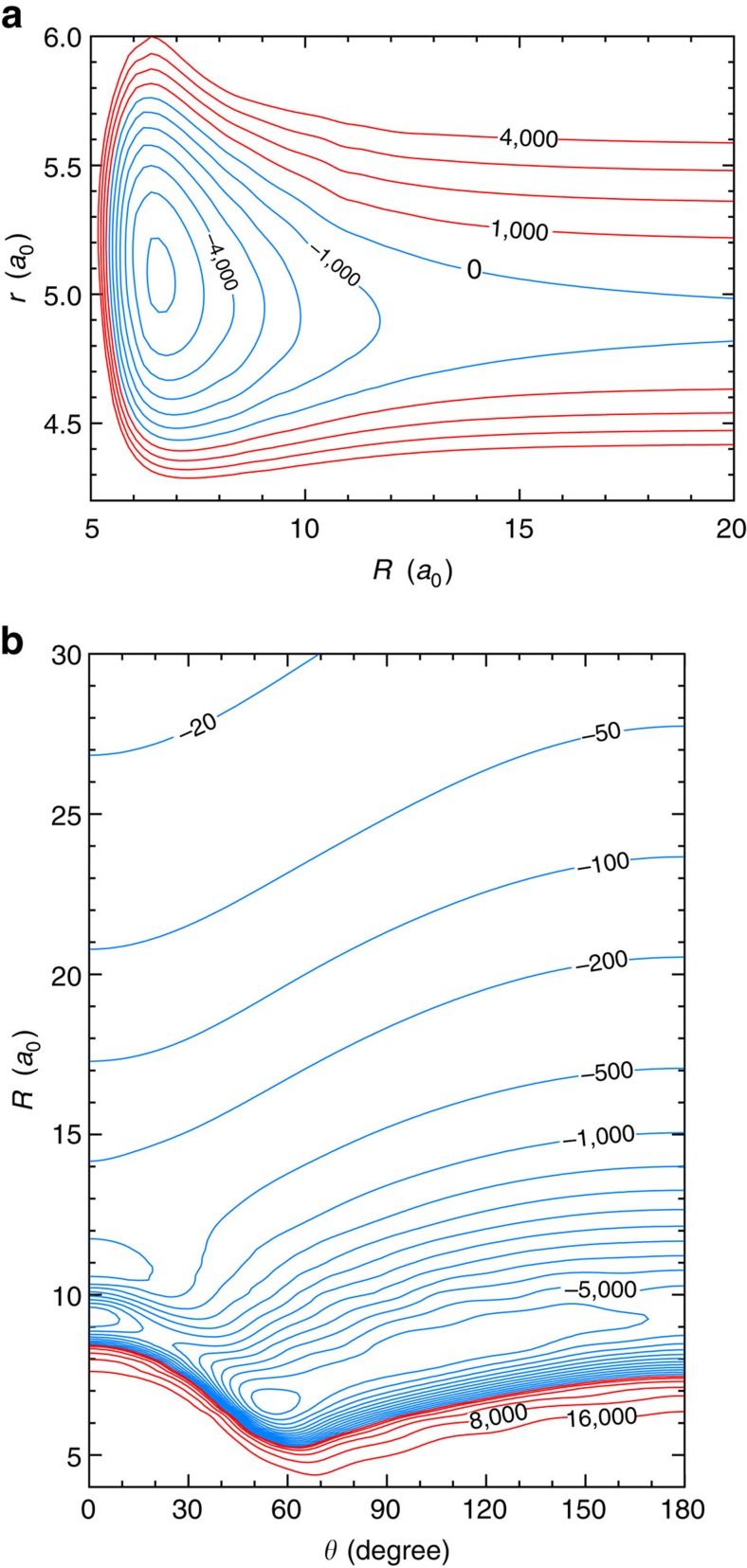
Potential energy. (**a**) Contour plot of the total PES for *θ*=55°. Contour energies are regularly spaced by 1,000 cm^−1^. (**b**) Contour plot of the three-body interaction energy for *r*=5 *a*_0_. Below −500 cm^−1^, contour energies are regularly spaced by 500 cm^−1^. Red contours correspond to positive energies, and blue to zero and negative energies.

The results of these calculations are shown in Fig. 1 along two dimensions in the Jacobi space. Figure 1a shows the existence of a relatively deep potential well in good agreement with the charge-transfer nature of the bonding within this ionic complex. Figure 1b reveals the existence of two minimal structures and two saddle points connecting these equilibrium structures. It also shows that the potential is strongly anisotropic. Although the Ba–Cl bond length is only slightly extended by the interaction with the calcium, we observe in Fig. 1a that the vibrational potential of BaCl^+^ is significantly modified by the latter interaction. This indicates there is a significant coupling between the vibration of BaCl^+^ and the other modes of motion. This coupling is expected to promote vibrationally inelastic collisions.

### Close-coupling calculation of the vibrational quenching rate

Calculation of the vibrational quenching rate on such a complex PES is normally done through the use of approximation techniques (see the Methods for details), which are not applicable at ultracold temperatures. Therefore, the use of the close-coupling method^8^ is compulsory, despite the fact that several other features of this system make the calculation tremendously intense—in fact, to our knowledge, no similar calculation has ever been performed before. Owing to the strong long-range and anisotropic potential, the small value of the rotational constant of BaCl^+^, and the large relative mass, the calculation must be performed to extraordinarily large distances (up to 2,000 *a*_*o*_) with matrices for a given value of *J* and parity that are as large as 10^4^ × 10^4^. Therefore, several theoretical and numerical improvements were required to make the calculations possible (see the Methods for details), including the development of a new scattering code using asynchronous task parallelization to calculate the vibrational quenching rates.

**Figure 2:**
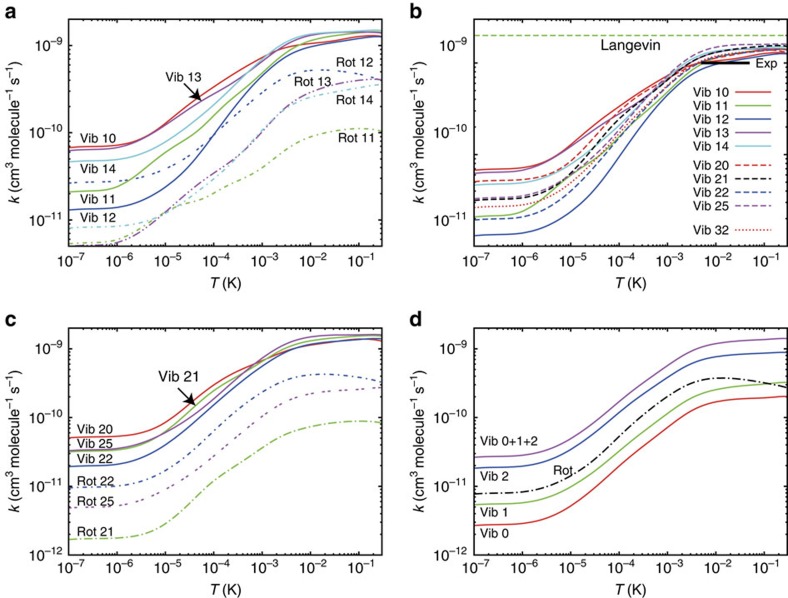
Calculated quenching rates. (**a**) Comparison between the vibrational and rotational quenching rate coefficients for BaCl^+^ in the initial states (*ν*=1, *j*=0,1,2,3,4). The first and second numbers designate, respectively, the initial vibrational and rotational quantum number of BaCl^+^. (**b**) Comparison between the vibrational quenching rate coefficients of several excited rovibrational levels (*ν*, *j*) of BaCl^+^ resulting from collisions with Ca with the experimental results and with the Langevin law. The first and second numbers designate, respectively, the initial vibrational and rotational quantum number of BaCl^+^. (**c**) Comparison between the vibrational and rotational quenching rate coefficients for BaCl^+^ in the initial states (*ν*=2, *j*=0,1,2,5). The first and second numbers designate, respectively, the initial vibrational and rotational quantum number of BaCl^+^. (**d**) Comparison between the vibrational and rotational quenching rate coefficients for BaCl^+^ in the initial state (*ν*=3, *j*=2). The label of each curve designates the final vibrational level.

Figure 2b shows the calculated vibrational quenching rate coefficients for a selected set of rovibrational states of BaCl^+^. A thick horizontal line represents the experimental measurement^4^ of the population averaged vibrational relaxation rate for *v*=1 and *v*=2, where the length of the line is representative of the energy range in the experiment. The ion-induced-dipole Langevin law^9^ is shown as a dashed line. The calculated values compare very well with the experiment, whereas the Langevin law is roughly double the experimental value. The close-coupling rate follows the Langevin law in the temperature domain of the experiment and departs from it at lower temperature—as discussed later, this departure is not due to quantum suppression^10^ as one might expect. In Figures 2a, 2c, 2d, the vibrational and rotational quenching are compared for several initial rotational levels belonging, respectively, to the vibrational levels *ν*=1, 2 and 3. The vibrational quenching is always larger than the rotational quenching. This very unusual result is due to the low value of the vibrational frequency of BaCl^+^ and the deep potential well, together yielding a strong coupling between many vibrational levels. This is in contrast with previously studied atom-diatom van der Waal neutral or ionic complexes^11^,^12^,^13^, where the potential well is usually not deep enough to couple even two different vibrational levels of the diatom. The only other possibility to obtain vibrational quenching comparable to rotational quenching is when the bond length of the complex is smaller than expected with a pure Van der Waals interaction, indicating the rise of chemical bonding induced by electron sharing between monomers. This is, for example, the case of the He-CH^+^ complex^14^.

### QDT calculation of the vibrational quenching rate

Despite its obvious utility, the close-coupling calculation does not lend itself to an intuitive understanding of the collision physics. Therefore, we have also performed a QDT calculation^15^,^16^,^17^ of BaCl^+^-Ca vibrational quenching, where the radial Schrodinger equation is solved for the long-range, isotropic *R*^-4^ induction term from large separation and matched with boundary conditions at short range describing the amplitude and phase of flux returning from the chemical bonding region *η*_*ℓm*_(*E*) and *δ*_*ℓm*_(*E*), respectively (see the Methods for details).

**Figure 3:**
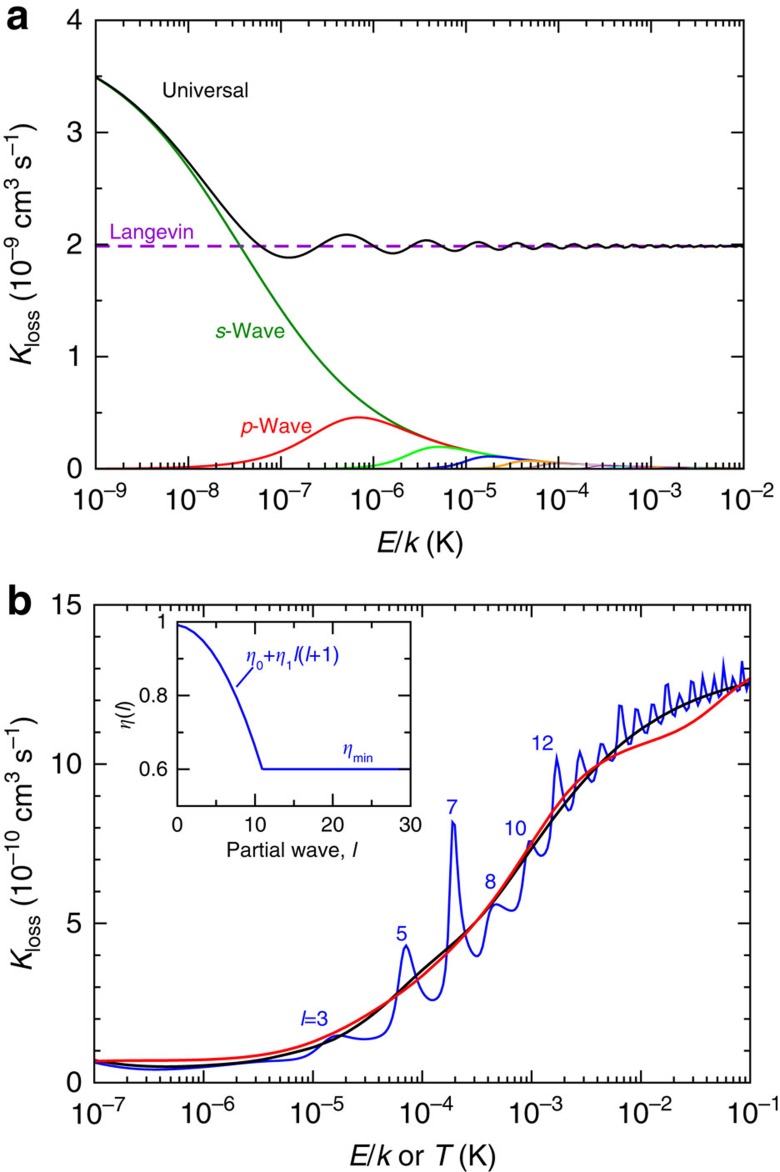
Quantum defect theory (QDT) of the Ca+BaCl^+^ collision. (**a**) Loss rate coefficients based on the QDT as functions of collision energy. The black solid line corresponds to the loss rate coefficient assuming universal or completely absorbing short-range boundary conditions. Coloured lines correspond to loss rates contributions from individual partial waves and its projections with *l*≤3. Finally, the dashed line is the loss rate coefficient found by the Langevin capture theory. (**b**) Loss rate coefficients *K*_*loss*_ as functions of collision energy or temperature for a partial-wave dependent QDT optimized to agree with the coupled-channels BaCl^+^(*v*=0, *j*=1)+Ca vibrational quenching rate coefficient. The blue curve shows *K*_*loss*_(*E*) as a function of collision energy, whereas the black curve shows the corresponding thermally averaged rate coefficient. The red curve corresponds to the *close-coupling results* ‘Vib 1 0' shown in Fig. 2. Shape resonances in *K*_loss_(*E*) are assigned by their partial wave *l*. The inset shows the short-range amplitude with parameters *η*_0_=0.9916, *η*_1_=−0.003 and *η*_min_=0.6. The short-range phase is *δ*_*ℓm*_(*E*)=0.34*π*.

Figure 3a shows the quenching rate coefficient in the so-called universal limit, where all collisions reaching short range lead to quenching, that is, *η*_*ℓm*_(*E*)=0. This rate agrees reasonably well with experiment and theory at the experimentally relevant energies, but dramatically overestimates the quenching rate at low energies. We thus conclude that the reduction in quenching rate at low energy is not due to quantum suppression as would be expected in systems with shorter ranged potentials^8^. Therefore, we match the QDT result to the close-coupling calculation by parameterizing the short-range boundary condition as *δ*_*ℓm*_(*E*)=*δ*_0_ and *η*_*ℓm*_(*E*)=*η*_0_+*η*_1_*l*(*l+*1) when 0≤*η*_min_≤*η*_*ℓm*_(*E*)≤1 and *η*_*ℓm*_(*E*)=*η*_*min*_ or 1 otherwise, where *η*_0_,*η*_min_, *η*_1_ and *δ*_0_ are determined by least-squares fitting (see the Methods for details). Figure 3b shows the good agreement between this fit and the close-coupling calculation and indicates that the suppression of the rate coefficient at low energies comes from lower quenching probabilities for small partial waves, as might be expected since the vibrational quenching is driven by asymmetry in the interaction potential.

### Statistical capture model for the vibrational quenching rate

In addition to these calculations, it is desirable to develop a model for vibrational quenching, which provides useful rules-of-thumb to both better understand the collision physics and guide future experiments. Unfortunately, the available models of vibrational quenching^18^ are not appropriate in this regime. The Landau–Teller model^19^, which is the most common model, is restricted to high collision energy. The only model available at low collision energy, owing to Dashevskaia and Nikitin^20^, cannot be applied to ionic systems as it makes the hypothesis that the long-range interaction potential is weak. Therefore, in order to rationalize our results, we introduce a statistical capture model following the recent work of Lara *et al.* on the ultra-low-temperature reactivity of D^+^+H_2_ reaction^21^. This model divides the quenching process into two steps: the formation of the collision complex through the long-range interaction potential followed by its fragmentation to produce a vibrationally quenched diatomic cation. This scheme relies on the statistical ansatz, which is valid for collisions involving deep intermediate wells and/or ultra low temperature. The capture process is described by the Langevin rate, whereas the probability of vibrational quenching is proportional to the number of accessible vibrational channels, which is roughly measured by the ratio *D*_e_/*ω*_e_, where *D*_e_ is the triatomic molecular ion dissociation energy and *ω*_e_ the diatomic vibrational frequency. Therefore, we define the statistical capture rate constant 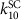, independent of the temperature, as:


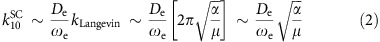


**Figure 4:**
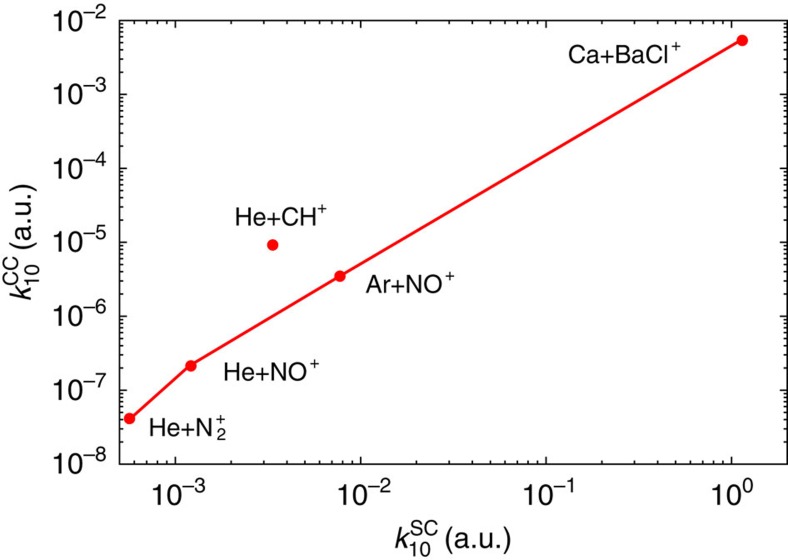
Close-coupling and statistical capture comparison. Comparison between the close-coupling vibrational quenching rate coefficients 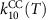 with the statistical capture rate 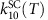 for five different colliding systems with the diatomic cation in the initial state (*ν*=1, *j*=0).

The validity of this model can be tested by comparing to known atom-ion quenching rates. Figure 4 shows a comparison of the statistical capture model to the quenching rate calculated from the Wigner threshold law for Ca-BaCl^+^ as well as several other species, which have been analysed by some of the authors^9^,^10^,^11^,^12^. Given the strong correlation with statistical capture model, we can create rules-of-thumb to aid future experiments realize efficient vibrational cooling. Namely, the vibrational quenching efficiency depends both on the strength of the long-range interaction potential, given by *α*, and on the density of states of the complex, given by *D*_e_/*ω*_e_. The increase of the state density increases the lifetime of the complex and facilitates vibrational quenching.

## Discussion

Among the possible explanations of the very large rate coefficients for the vibrational quenching, which is even larger than those for rotational quenching, one could be tempted to think about quasi resonant vibration-rotation energy transfer. This type of transfer is well documented for neutral systems like the collisions of Li_2_ and HF with neutral noble gases at high temperatures^22^ and has also been predicted theoretically to occur for the neutral He-H_2_ collision at very low temperatures^23^. Quasi resonant vibration-rotation energy transfer, however, is expected for molecules in highly excited initial rotational states and takes place when the rapidly rotating diatom is stretched to its outer turning point and collinear with the atom^24^. In the present case, the calculated vibrational quenching rate coefficients are already unusually large for the lowest *j*=0-5 rotational quantum numbers of BaCl^+^. This mechanism then cannot be at play.

The calculations presented here confirm the observation of efficient vibrational cooling in the Ca-BaCl^+^ system and provide a simple means to predict new systems that will exhibit efficient cooling. As all laser coolable atoms exhibit large polarizabilities, the only requirement on future experiments is to choose molecular ions that are strongly bound. This requirement is not particularly restrictive as other requirements of the effort, for example, non-reactivity with the ultracold atom and the existence of a large dipole moment, are typically only satisfied for strongly bound molecular ions. Therefore, sympathetic cooling of molecular ions with ultracold atoms appears to be poised to provide a generic and robust route to harness the potential of molecular structure for science and technology.

## Methods

### PES calculation

The electronic ground state of the CaBaCl^+^ complex is a ^1^A′ state. In preliminary computations, we found that the wavefunction is dominantly described by a single electronic configuration. Therefore, the interaction energy between Ca and BaCl^+^ entities was computed using the coupled cluster method based on single and double electronic excitations and a perturbative treatment of triple excitations, CCSD(T). The counterpoise procedure was used to correct the interaction energy for the basis set superposition error. All electronic calculations were performed with the MOLPRO suite of programmes^7^. We used the def2-QZVPPD basis set^25^, which was augmented by diffuse functions. For chlorine, this results on (21s,15p,5d,2f,1g) primitives, which were contracted into (10s,7p,5d,2f,1g) and for calcium (24s,18p,6d,3f) contracted into (11s,6p,4d,3f) basis sets. For the barium atom, only the valence electrons were described by the (8s,8p,5d,3f) primitives contracted into (7s,5p,3d,3f), whereas we used also the quasi-relativistic 10-valence-electron pseudopotential ECP46MWB^26^ for the 46 inner electrons.

In this manner, the interaction energy was calculated on a 3D grid of points in the Jacobi coordinates space. The 3D grid is a direct product of the three 1D grids spanning the Jacobi coordinates *r*, *R* and *θ*. Here, *r* represents the BaCl^+^ bond length, *R* the distance between Ca and the centre of mass of BaCl^+^, and *θ* the angle between **r** and **R**, with the linear structure Ba-Cl-Ca corresponding to *θ*=0°. For the *r* bond length, we selected 15 values ranging from 3.85 to 6.05 *a*_0_. The distance *R* took 50 values varying from 4 to 50 *a*_0_, with a variable step increasing from 0.2 to 10 *a*_0_. The *θ* angle was uniformly distributed from 0° to 180° by step of 10°.

The evolution of the potential energy curve (PEC) and the dipole moment of the isolated BaCl^+^ diatomic along the *r* coordinate were calculated with the Davidson corrected multi-reference configuration interaction (MRCI+Q) method^27^ using the molecular orbitals calculated by the complete active space self consistent field (CASSCF) technique^28^. State-averaged CASSCF calculations (over 8 states with equal weight) were performed, along with 12 active orbitals and 6 active electrons. The basis set and the core pseudopotential defined above were used. A grid of 26 points from 3.4 to 20 *a*_0_ was calculated.

The parallel and perpendicular static polarizabilities of BaCl^+^ were calculated using the finite-field procedure. For that purpose, we computed the ground-state CASSCF/MRCI+Q energy, the CASSCF calculations being performed with the ground-state orbitals only. The polarizabilities were calculated on a grid of 17 points from 3.7 to 8 *a*_0_. The ionization energy of BaCl^+^ was obtained as the energy difference between the energy of BaCl^+^ and of BaCl^++^, both taken in their respective electronic ground state. The potential of BaCl^++^ was calculated at the CASSCF/MRCI+Q level where the CASSCF was state-averaged on two states and using 5 active electrons distributed in 12 active orbitals.

The functional form *V*(*r,R,θ*) of the PES is defined as the sum of the interaction energy *V*_I_ between Ca and BaCl^+^ and the potential of the isolated diatomic BaCl^+^, *V*_BaCl_:


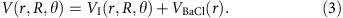


The potential *V*_BaCl_ is obtained by cubic splines interpolation of the *ab initio* data. The functional form of the interaction energy *V*_I_(*r,R,θ*) is defined as the sum of the short-range *V*^S^ and long-range *V*^L^ contributions, combined with the switching function *S*(*R*):


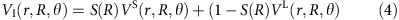


with the short-range function defined by


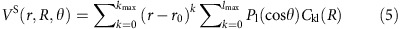


where *P*_l_(cos*θ*) are normalized Legendre polynomials. The coefficients *C*_kl_(*R*) have been first calculated at the points of the *R*-grid by the linear least squares method, using *k*_max_=10, *l*_max_=10 and *r*_0_=5 *a*_0_. Then, a cubic splines interpolation is used to obtain the coefficients for any value of *R* inside the limits of the *R*-grid.

The switching function, which ensures a smooth connection between the long-range and the short-range functions, is defined by


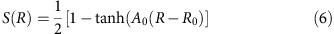


with *R*_0_=20 *a*_0_, and *A*_0_=0.14 *a*_0_^−1^.

The long-range part is defined as a the sum of the leading terms of induction and dispersion energies^29^,


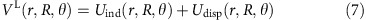



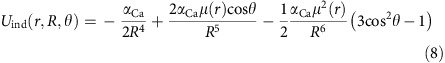



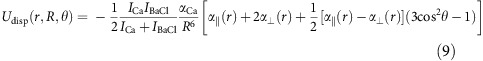


where the dipole moment *μ*, the parallel *α*_||_ and perpendicular *α*_⊥_ polarizabilities of BaCl^+^ were obtained by polynomial interpolation of the *ab initio* data


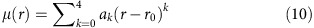



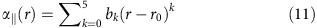



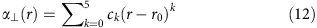


and where the coefficients *a*_*k*_, *b*_*k*_ and *c*_*k*_ have been determined by the linear least square method. The polarizability of calcium^30^ is *α*_Ca_=168.71 *a*_0_^3^, the ionization energy of calcium^28^ *I*_Ca_=6.113 eV, and the ionization energy of BaCl^+^ *I*_BaCl_=13.9 eV. Let us note that, although using the long-range interaction model without any dependence in the internal coordinates is a customary approximation, we have chosen here to take in account the vibrational coordinate because this work focuses on vibrational quenching.

The spectroscopic properties of the fitted diatomic potential for BaCl^+^(X^1^Σ^+^) are *r*_e_=4.899 *a*_0_, *D*_e_=39,278 cm^−1^, *ω*_e_=333.8 cm^−1^ and *ω*_e_*x*_e_=0.837 cm^−1^. These values are close to the theoretical data previously reported^31^. The dipole moment calculated at the equilibrium bond length is *μ*_e_=8.927 Debye. This calculation was done with the origin of coordinates fixed at the centre of mass, using the average atomic masses.

We display in Fig. 1a the contour plot of the total potential energy along the *r* and *R* coordinates, and in Fig. 1b the contour plot of the interaction energy along the *R* and *θ* coordinates. The plot in Fig. 1a shows the existence of a relatively deep potential well in good agreement with the charge-transfer nature of the bonding within this ionic complex. Figure 1b reveals the existence of two minimal structures and two saddle points connecting these equilibrium structures. It also shows that the potential is strongly anisotropic. The existence of these stationary points on the ground potential of the Ca-BaCl^+^ system was checked by *ab initio* geometry optimizations where all coordinates were kept free. The Jacobi coordinates of the global minimum of *V*(*r,R,θ*) are *r*=5.08 *a*_0_, *R*=6.54 *a*_0_ and *θ*=54.7°. This means that the calcium atom is bonded to the chlorine end of BaCl^+^, with bond length *r*_CaCl_=5.34 *a*_0_ and angle *θ*_CaClBa_=87.1°. The electronic dissociation energy is *D*_e_^T^=6,263.8 cm^−1^. If we consider the interaction potential alone, the coordinates of the global minimum are then *r*=5.41 *a*_0_, *R*=6.49 *a*_0_ and *θ*=52.8°, and the corresponding dissociation energy is 7,441.9 cm^−1^.

Although the Ba-Cl bond length is only slightly extended by the interaction with the calcium, we observe in Fig. 1a that the vibrational potential of BaCl^+^ is significantly modified by the latter interaction. This indicates there is a significant coupling between the vibration of BaCl^+^ and the other modes of motion. This coupling is expected to promote vibrationally inelastic collisions.

At long range, the induction term that scales as *R*^−5^ is the leading *anisotropic* interaction. The angular dependence (see equation 6) of this long-range interaction potential tends to align the three atoms Ca-Ba-Cl. Thus, at low collision energies, we expect a propensity for the alignment of the reactants, resulting in pendular states of BaCl^+^ around the linear structure. It is only for *R*<10 *a*_0_ that the propensity for the linear structure begins to disappear gradually with decreasing *R*. The minimum of the potential energy of the linear structure is found at *r*=4.94 *a*_0_ and *R*=9.50 *a*_0_, with a dissociation energy of 5,387.6 cm^−1^. This minimum is a saddle point, as bending the Ca-Ba-Cl structure leads to the global minimum.

### Close-coupling calculations

The use of the long established close-coupling method^6^ to study the dynamics of atom-diatom rovibrational inelastic collisions seems at first to be a simple task. However, the deep potential well of the system and both the very small vibrational and rotational quanta^29^ of BaCl^+^ make the size of such calculations tremendous and one could be tempted to consider the use of more approximate alternative approaches like the infinite order sudden approximation^32^ or the coupled states approximation^33^,^34^ methods. Unfortunately, a prerequisite for use of the infinite order sudden approximation is that the rotational spacing of the diatom has to be negligible compared with the collision energy, which would become true above 10 cm^−1^, but is not satisfied in the energy domain of the ultracold experiment. Further, this approximation requires that the well depth be small compared with the collision energy, such that couplings with the closed channels remain small. With a well depth of 7,442 cm^−1^, the use of this approximation is then restricted to very high collision energies for this system. Similarly, the coupled states approximation method is expected to be valid for rotor states whose relative kinetic energy is large compared with the well depth; it is therefore not applicable to Ca-BaCl^+^ at very low collision energy.

The use of the close-coupling method then appears to be compulsory for this collision, despite the fact that several other features of this system that make such calculations especially intense. First, the strong angular anisotropy of the potential and the small value of the rotational constant of BaCl^+^ result in the coupling of many rotational levels. Second, the strong long-range potential requires propagation of the calculation to very long intermolecular distances. Third, the large relative mass necessitates many values of the total angular momentum quantum number *J* to reach convergence. The size of the matrices that have to be propagated for a given value of *J* and parity can then be as big as 10^4^ × 10^4^ (above a collision energy of 1 cm^−1^), which make the calculations unusually heavy, even at very low collision energy.

Therefore, several theoretical and numerical improvements were required to make the calculations possible. First, the vibrational coupling matrices 〈*ϕ*_*vj*_|(*r*-*r*_0_)^*k*^|*ϕ*_*v*′*j*′_〉, where *ϕ*_*νj*_ denotes the asymptotic rovibrational eigenfunctions, were calculated once and stored before the close coupling calculation. Thus, the large vibrational quadrature (about 100 points) necessary to compute these coupling terms was reduced to a summation over 11 terms (*k* runs from 0 up to 10) in the close-coupling calculation. Second, the calculation of the cross-sections for a single collision energy, even the lowest one, converged as a function of the total angular momentum, takes several hundred of hours of CPU time and reaches quickly (around a collision energy of 1 cm^−1^) a thousand of hours. Therefore, we developed an MPI version of our Newmat code^35^ using asynchronous task parallelization. The elementary task is the propagation of the wavefunction at one particular collision energy. The MPI code distributes N tasks over M processors. Because the tasks are independent, this parallelization scheme requires no overhead and feeds efficiently all the processors.

We included 25 rotational levels in each of the 19 vibrational levels used to perform the calculations. The propagation step size was taken to be 0.015 *a*_0_ and the maximum distance of propagation was 400 *a*_0_. The calculations were performed in the collision energy interval (10^−6^, 10^0^) cm^−1^. The relative convergence criterion of the inelastic cross-sections as a function of the total angular momentum was taken to be 0.1% for the lowest energies, 1% for intermediate energies and 5% for the highest energies around 1 cm^−1^.

### Quantum defect theory

Despite its obvious utility, the close-coupling calculation does not readily lend itself to an intuitive understanding of the collision physics. Therefore, we have also developed a QDT for scattering between BaCl^+^ and Ca based on solutions of a single radial Schrödinger equation where the potential between the molecular ion and neutral atom is dominated by its longest-ranged and isotropic induction potential, *V*(*R*)=−*C*_4_/*R*^4^. At *R*=*R*_short_, where *R*_short_ is a characteristic short-range separation defined more precisely below, these solutions are uniquely specified by a boundary condition with a limited number of parameters that summarize the reflections (elastic collisions) and absorption (quenching collisions) in the complex, anisotropic and possibly chaotic evolution at short separations. By carefully choosing the collision-energy and partial-wave dependence of the boundary condition, we reproduce the temperature dependence of the rate coefficients for rovibrational relaxation as obtained by close-coupling calculations. The behaviour of these boundary conditions gives a simple, intuitive picture of the collision processes.

The long-range induction potential is much longer ranged than the van der Waals potential between two neutral atoms. As a result, many partial waves contribute to the rates; in fact only for temperatures of 100 nK are collisions *s*-wave dominated. Nevertheless, as we will show below, even for mK collision energies quantum mechanical effects profoundly affect the scattering of BaCl^+^ and Ca and play an important role in the description of the interplay between inelastic and elastic collisional processes.

In our QDT, we numerically solve the single-channel radial Schrödinger equation for collision energy *E*, partial wave *l* and projection *m* from separation *R*=*R*_short_ to ∞ with boundary condition 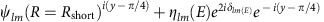, where *y*=*R*_4_/*R* and
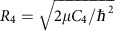
. The functions exp(±*i*[*y*−*π*/4]) can be recognized as Wentzel–Kramers–Brillouin solutions of a −*C*_4_/*R*^4^ potential at zero collision energy and partial wave. The short-range amplitude 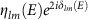 with real-valued functions *η*_*ℓm*_(*E*) and *δ*_*ℓm*_(*E*) determines the flux returning from the chemical bonding region where all three atoms are separated by no more than *R*_short_<<*R*_4_; we use *R*_short_≈30*a*_0_, just outside the separations where the electron wave functions have significant overlap. The short-range amplitude, in principle, are determined by the close coupling simulations. Flux conservation requires that 0≤*η*_*ℓm*_(*E*)≤1, where *η*_*ℓm*_(*E*)=0 corresponds to the case where no flux is returned from short range and *η*_*ℓm*_(*E*)=1 corresponds to the case where everything is reflected back. The phase *δ*_*ℓm*_(*E*) describes the relative phase shift of the returning flux. In the limit *R*→∞, the wavefunction approaches 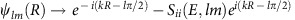, where 
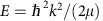
, *k* is the relative collision wavevector, and *S*_*ii*_(*E*,*lm*) is the diagonal S-matrix element from which elastic and total inelastic rate coefficients can be determined. In fact, the total inelastic rate coefficient is 
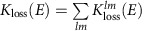
 with 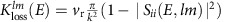, where *v*_r_ is the relative velocity. Here, we have used flux conservation or the unitarity of the *S*-matrix to rewrite the loss rate coefficient solely in terms of the diagonal *S*-matrix element, *S*_*ii*_(*E*,*lm*). At ultracold temperatures, only a few partial waves *l* contribute as for higher *l* the centrifugal barrier prevents the atom and molecule from approaching each other and 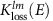
rapidly goes to zero with increasing *l*.

The −*C*_4_/*R*^4^ potential is the largest energy scale at *R*=*R*_short_ and, consequently, we initially assume that *η*_*ℓm*_(*E*) and *δ*_*ℓm*_(*E*) are independent of collision energy *E*, partial wave *l* and projection *m*. In fact, ultracold reactions between neutral KRb and K, with a long-range van der Waals dispersion potential, forming K_2_ have been successfully modelled^36^ with *η*_*ℓm*_(*E*)<<1. It is then natural to first study the universal limit of *η*_*ℓm*_(*E*)=0, that is, all short-range collisions lead to quenching, for our induction potential. Figure 3a shows the universal loss rate coefficient as a function of *E* from *E*/*k*=1 nK to 0.01 K. At very low energy, the rate is dominated by *s*-wave scattering and is a decreasing function of *E*. At higher energy, other partial waves contribute creating a rate coefficient that weakly oscillates around the rate coefficient predicted by Langevin capture theory. Although the QDT universal approximation roughly agrees with both the experimental determination and the close-coupling calculation for energies above 1 mK, the universal result dramatically over estimates the quenching rate for temperatures below 1 mK.

Therefore, in order to reproduce the temperature dependence of the coupled-channel calculation with QDT, we must use a collision-energy- and partial-wave-dependent short-range amplitude 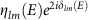. For practical purposes, the number of parameters that describe *η*_*ℓm*_(*E*) and *δ*_*ℓm*_(*E*) must be limited and, here, we use the fact that QDT aims to only describe cross-sections over a small range of collision energies and limited number of partial waves. In our case, we need to represent collision energies below 0.1 K and partial waves up to 35. We then observe that at *R*=*R*_short_ the rotational energy 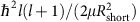 for *l*=35, although much smaller than the 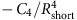 potential energy, is much larger than the relevant range of *E*. Hence, we can assume that the short-range parameters only depend on *l*. In fact, we further limit the parameterization to *δ*_*ℓm*_(*E*)=*δ*_0_ and *η*_*ℓm*_(*E*)=*η*_0_+*η*_1_*l*(*l*+1) when 0≤*η*_min_≤*η*_*ℓ*m_(*E*)≤1 and *η*_*ℓm*_(*E*)=*η*_min_ or 1 otherwise. There are four free variables *η*_0_, *η*_min_, *η*_1_ and *δ*_0_.

Figure 3b shows the best fit of QDT to the BaCl^+^(*v*=1,*j*=0)+Ca vibrational quenching rate coefficient. The figure shows both its energy dependence and the corresponding thermally averaged value. The partial wave dependence of *η*_*ℓm*_(*E*) is shown in the inset. The energy-dependent rate coefficient is less than 10^−10^ cm^3^ s^−1^ for *E*/*k*≈1 μK. For these energies only *l*=0 and 1 contribute, *η*_*ℓm*_(*E*) is close to one, and losses are suppressed. The rate coefficient then increases, interrupted by multiple resonances, to a rate coefficient of about one half of the Langevin rate coefficient near *E*/*k*≃0.1 K, where nearly 35 partial waves contribute and *η*_*ℓm*_(*E*)=*η*_min_. In the thermally averaged rate coefficient, the resonances, except for those below *T*=100 μK, have washed out. Rate coefficients for other rovibrational levels (*v*,*j*) can be found in a similar manner and lead to slightly different parameters *η*_0_, *η*_min_, *η*_1_ and *δ*_0_.

**Figure 5:**
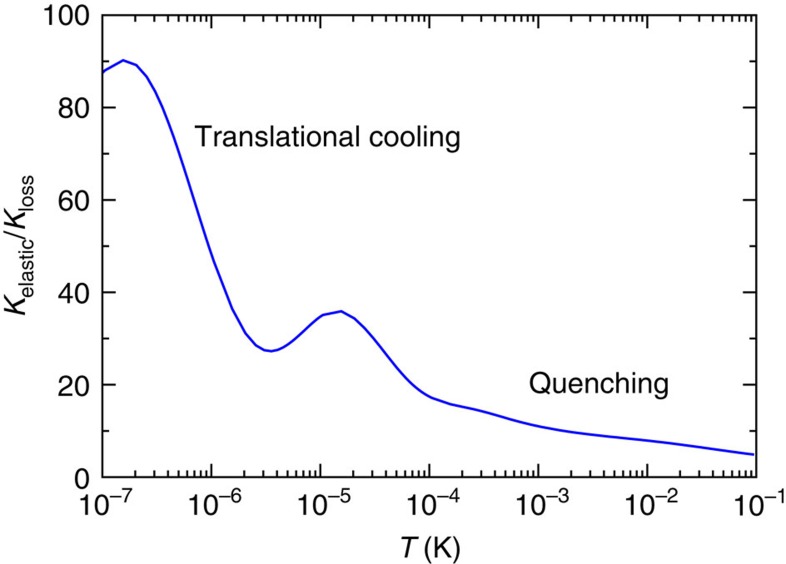
Relative rate dependence on partial wave. Ratio of the thermalized elastic and loss rate coefficients, *K*_elastic_/*K*_loss_, as function of temperature for a partial-wave-dependent optimized QDT of BaCl^+^+Ca. Parameters as in Fig. 3b.

The location and origin of the series of resonances explains much of our successful fit. They are shape resonances behind the even partial-wave centrifugal barriers. Analysis of the analytic *E*=0 solution of the −*C*_4_/*R*_4_ potential with short-range amplitude *η*_*ℓm*_(*E*=0)=1 and *l*-independent phase *δ*_*ℓm*_(*E*=0) has shown that if an *E*=0 bound state occurs for partial wave *l* then such bound state also exist for the ..., *l*-4, *l*-2, *l*+2, *l*+4, ... partial waves. This condition is only satisfied for two values of the short-range phase *δ*_0_, one corresponding to all even partial waves, one to the odd waves. For different values of *δ*_0_ as well as *η*_*ℓm*_(*E*)<1, these bound states become shape resonances where the inelastic loss is resonantly enhanced. For BaCl^+^+Ca collisions with its *η*_*ℓm*_(*E*)≈*η*_0_≈1 for small partial waves, we find that near the optimal *δ*_0_ resonances occur for odd partial waves and, in particular, near *E*/*k*=10 μK the rate coefficient is sensitive to the location of the *l*=3, *f*-wave resonance. For *E*/*k*>0.5 mK, where *l*≥8 wave collisions become prominent, the amplitude *η*_*ℓm*_(*E*) deviates sufficiently from one, such that resonances from even partial wave collisions are observed. Finally, for the optimal conditions, the absolute value of the elastic scattering length *a* for s-wave BaCl^+^+Ca collisions is larger than the natural length scale of the *C*_4_ potential. In this case, the finite Wigner-threshold prediction for the loss rate coefficient is only reached for collision energies below *E*/*k*=1 nK not shown in Fig. 3b. Figure 5 predicts the ratio of the QDT elastic and loss rate coefficient as a function of temperature. Ratios much larger than one indicate that the kinetic energy in the centre-of-mass motion of the molecular ion is more efficiently relaxed than its vibrational energy. For example, for BaCl^+^ ions at *T*=0.1 K, the ratio is close to one. We anticipate two cooling stages for BaCl^+^ ions in collisions with ultracold and highly polarizable Ca atoms. In the first stage cooling or relaxation of the rovibrational states occurs, as the small ratio *K*_elastic_/*K*_loss_ implies that translational cooling in this region will be inefficient. Only when the molecules are in the lowest rovibrational state, the second stage, will elastic collisions cool the external motion of the molecules.

**Figure 6:**
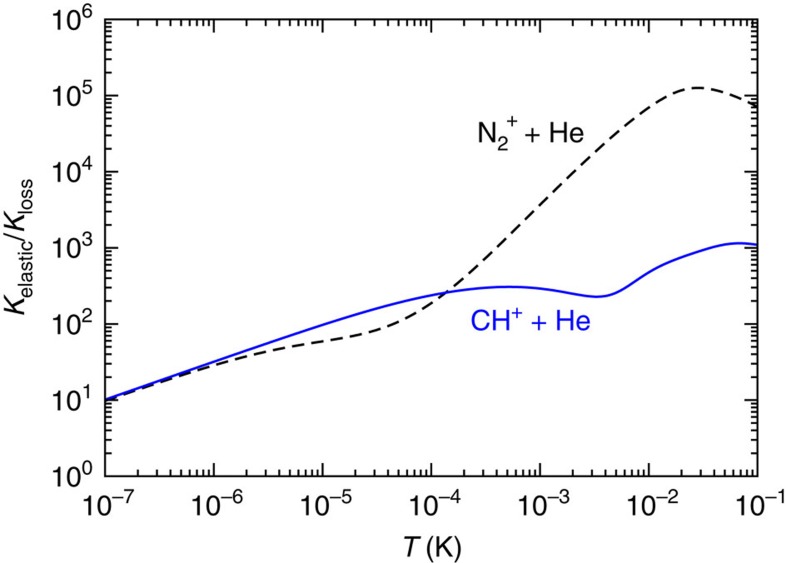
Quantum defect theory (QDT) for previously studied systems. Ratio of the thermalized elastic and loss rate coefficients, *K*_elastic_/*K*_loss_, as function of temperature for the N_2_^+^+He (dashed line) and CH^+^+He (solid line) systems using optimized QDT.

Traditional cooling schemes often involve collisions of ionic molecules with He gas at cryogenic temperatures. Here, we analyse the ratio *K*_elastic_/*K*_loss_ for two ionic systems He-N_2_^+^ and He-CH^+^ to demonstrate a different cooling mechanism than for cooling with Ca. First, we fit the He-N_2_^+^ and He-CH^+^ vibrational quenching rate coefficients obtained in the coupled-channel calculations^10^,^12^ to our QDT theory with partial wave-dependent short-range parameters. The parameters of our best fit are *η*_0_=1–5.7*10^−6^, *η*_1_=−2*10^−6^, *δ*_*ℓm*_(*E*)=0.76***π*** for He-N_2_^+^ and *η*_0_=1–7*10^−3^, *η*_1_=0 and *δ*_*ℓm*_(*E*)=0.06*π* for He-CH^+^. Figure 6 shows the temperature-dependent ratio *K*_elastic_/*K*_loss_ for these two systems. It is evident that for both systems the elastic rate coefficient is much larger than that of the inelastic processes. Hence, for a molecular ion in a given excited rovibrational level its translational motion will be cooled first. Occasionally, an inelastic process relaxes this internal state at the cost of rapid increase of the translational temperature. Elastic collisions will then start the cooling all over again.

### Statistical capture model

To rationalize our results, we compare in Table 1 five collisions involving a diatomic cation and a rare gas. We report in this table the dissociation energy of the complex, the vibrational frequency of the diatomic cation, the relative mass of the system, the polarizability of the rare gas atom and the imaginary part of the scattering length *β*_10_ associated with the collisional vibrational quenching of the lowest rotational level of the first excited vibrational level of the diatomic cation. In the Wigner regime, the quenching rate coefficient is independent of the temperature and directly proportional to *β*_10_:


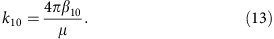


The values reported in the table were obtained from our close-coupling calculations for the collision energy of 10^−6^ cm^−1^, where only the *s*-wave contributes to the collision. The propagation at such low collision energy has to be performed up to very large values of the intermolecular separation coordinate. For Ca-BaCl^+^, this maximum distance had to be extended to 2,000 *a*_0_. Because of the long-range ion induced dipole potential, which furthermore requires applying modified effective range theory^37^, the use of the scattering length approximation is limited to even lower collision energies. The usual formulas linking the real and imaginary parts of the scattering length with the scattering S matrix are in this case valid only for collision energies lower that 10^-10^ cm^−1^. The properties of the Ca-BaCl^+^ system, namely a very low vibrational frequency, a deep potential well, a large value of the three quantities—equilibrium diatomic distance, relative mass and atomic rare gas polarizability—are seen to differ strongly from those of the other systems. But what makes BaCl^+^ especially singular is the unusually small value of its vibrational frequency (330 cm^−1^)—compared with ∼2,000 cm^−1^ for the other diatoms considered. As seen in Table 1, the vibrational quenching scattering length *β*_10_ monotonously increases as a function of the triatomic ion well depth *D*_e_ and also as a function of *D*_e_ divided by the diatom vibrational frequency *ω*_e_. This correlation was noticed long ago by many authors^18^ and the Landau–Lifshitz probability for vibrational quenching of neutral molecules used by Dashevskaya and Nikitin^18^, for example, is mainly dependent on both parameters *ω*_e_ and *D*_e_. At very low collision energy, the long-range part of the interaction potential plays a major role, whereas the ratio *D*_e_/*ω*_e_ offers a crude measurement of the efficiency of the vibrational energy redistribution. Therefore, we define the statistical capture rate constant 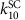, independent of the temperature, by simply multiplying the Langevin rate coefficient by this ratio *D*_e_/*ω*_e_:


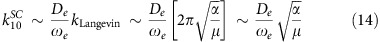


**Figure 7:**
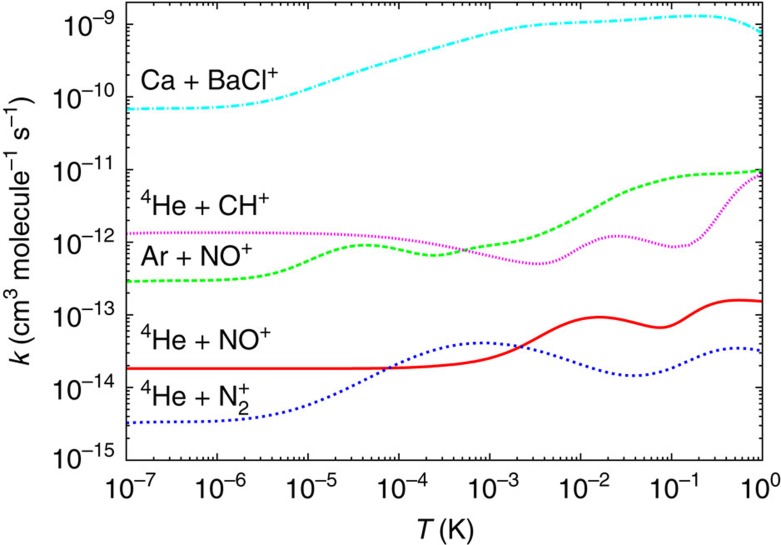
Comparison of quenching rates for present and previously studied systems. Close coupling vibrational quenching rate coefficients *k*_10_^CC^(*T*) for five different colliding systems with the diatomic cation in the initial state (*ν*=1,*j*=0).

This relation can be formally introduced through a capture statistical approach, which was presented in the body of this manuscript. This simple relation makes the vibrational quenching proportional not only to the *D*_e_/*ω*_e_ ratio but also to the polarizability of the ultra cold atom and to the relative mass of the system. These two last results were expected as they express the dependence of the quenching efficiency on the strength of the long-range interaction potential and on the density of states of the complex. The increase of the state density increases the lifetime of the complex and facilitates vibrational quenching. We notice, however, that the ^4^He-CH^+^ system seems to behave differently than the other systems. This is effectively what can be seen on Fig. 7 where the Close coupling rate coefficients were reported for these five systems in the [10^−7^, 1] Kelvin interval. The ^4^He-CH^+^ rate coefficient is the only one for which the rate coefficient decreases instead of increasing above the Wigner regime. This behaviour was shown in our paper dedicated to this system^12^ to be due to virtual state scattering^38^. This is indeed the only one of these five systems for which the real part of the scattering length is negative. Virtual state scattering increases the close coupling value of the quenching rate coefficient and our simple model then allows predicting a lower bound for the zero temperature limit for all the studied systems.

## Additional information

**How to cite this article:** Stoecklin, T. *et al.* Explanation of efficient quenching of molecular ion vibrational motion by ultracold atoms. *Nat. Commun.* 7:11234 doi: 10.1038/ncomms11234 (2016).

## Figures and Tables

**Table 1:** Comparison of some of the main features of several atom-cationic diatom vibrationally inelastic collisions in the limit of zero temperature.

**System A-BC**^**+**^	***ω***_**e**_**(cm**^**−1**^**)**	***B***_**rot**_**(cm**^**−1**^**)**	***R***_**e**_**(*****a***_**0**_**)**	***D***_**e**_**(cm**^**−1**^**)**	***μ*** **(g mol**^**−1**^**)**	***β***_**10**_ **(*****a***_**0**_**)**	***α*** **(Å**^**3**^**)**	**Reference**
^4^He-N_2_^+^	2,207	1.93	6.08	*−*84*.5*	3.50	2.5 10^−4^	0.2	ref. 12
^4^He-NO^+^	2,376	1.997	5.26	−195.4	3.53	1.38 10^−3^	0.2	ref. 13
^4^He-CH^+^	2,380	14.24	4.10	−513.6	3.06	5.3 10^−2^	0.2	ref. 14
Ar-NO^+^	2,376	1.997	5.86	−980.4	17.1	1.1 10^−1^	1.64	ref. 11
Ca-BaCl^+^	334	0.09	6.49	−7,442	40	3.26 10^2^	22.8	

*β*_10_ is the value of the Close Coupling imaginary part of the scattering length for the collisional vibrational quenching rate coefficient of the (*ν*=1, *j*=0) state of the cationic diatom, computed at the collision energy of 10^-6^ cm^−1^. For each system, the relative mass *μ*, the equilibrium intermonomers distance *R*_e_, the well depth *D*_e_ of the triatomic ABC^+^ complex, the diatom vibrational frequency *ω*_e_, the diatom rotational constant *B*_rot_ and the polarizability of the impinging atom are reported.

## References

[b1] Carr L. D. *et al.* Cold and ultracold molecules: science, technology and applications. N. J. Phys. 11, 055049 (2009).

[b2] Hutzler N. R., Lu H. I. & Doyle J. M. The Buffer Gas Beam: an intense, cold, and slow source for atoms and molecules. Chem. Rev. 112, 4803–4827 (2012). 22571401 10.1021/cr200362u

[b3] Hansen A. K. *et al.* Efficient rotational cooling of coulomb-crystallized molecular ions by a helium buffer gas. Nature 508, 76–79 (2014). 24670662 10.1038/nature12996

[b4] Campbell W. C. *et al.* Time-domain measurement of spontaneous vibrational decay of magnetically trapped NH. Phys. Rev. Lett. 100, 083003 (2008). 18352622 10.1103/PhysRevLett.100.083003

[b5] Rellergert W. G. *et al.* Evidence for sympathetic vibrational cooling of translationally cold molecules. Nature 495, 490–495 (2013). 23538830 10.1038/nature11937

[b6] Hudson E. R. Method for producing ultracold molecular ions. Phys. Rev. A 79, 032716 (2009).

[b7] Werner H.-J. *et al.* MOLPRO http://www.molpro.net (2012).

[b8] Arthurs A. & Dalgarno A. The theory of scattering by a rigid rotator. Proc. R. Soc. Lond. Ser. A 256, 540–551 (1960).

[b9] Levine R. D. & Bernstein R. B. Molecular Reaction Dynamics and Chemical Reactivity Oxford Univ. (1987).

[b10] Côté R., Heller E. J. & Dalgarno A. Quantum suppression of cold atom collisions. Phys. Rev. A 53, 234–241 (1996). 9912879 10.1103/physreva.53.234

[b11] Halvick P., Stoecklin T., Lique F. & Hochlaf M. Explicitly correlated treatment of the Ar-NO^+^ cation. J. Chem. Phys. 135, 044312 (2011). 21806124 10.1063/1.3614502

[b12] Stoecklin T. & Voronin A. Strong isotope effect in ultracold collison of N_2_^+^ (*v*=1, *J*=0) with He: a case study of virtual-state scattering. Phys. Rev. A 72, 042714 (2005).

[b13] Stoecklin T. & Voronin A. Vibrational and rotational cooling of NO^+^ in collisions with He. J. Chem. Phys. 134, 204312 (2011). 21639445 10.1063/1.3590917

[b14] Stoecklin T. & Voronin A. Vibrational and rotational energy transfer of CH^+^ in collisions with ^4^He and ^3^He. Eur. Phys. J. D 46, 259–265 (2008).

[b15] Idziaszek Z. & Julienne P.S. Universal rate constants for reactive collisions of ultracold molecules. Phys. Rev. Lett. 104, 113202 (2010). 20366474 10.1103/PhysRevLett.104.113202

[b16] Kotochigova S. Dispersion interactions and reactive collisions of ultracold polar molecules. N. J. Phys. 12, 073041 (2010).

[b17] Gao B. Quantum defect theory for -1/r^4^-type interactions. Phys. Rev. A 88, 022701 (2013).

[b18] Smith I. W. M. Collisional energy transfer, intramolecular vibrational relaxation and unimolecular reactions. J. Chem. Soc. Faraday. Trans. 93, 3741–3750 (1997).

[b19] Ferguson E. E. Vibrational quenching of small molecular ions in neutral collisions. J. Phys. Chem. 90, 731–738 (1986).

[b20] Dashevskaya E. I. & Nikitin E. E. Quantum Suppression and enhancement of the quasiclassical Landau-Lifshitz matrix elements: Application to the inelastic H_2_-H scattering at ultralow energies. Phys. Rev. A. 63, 012711 (2000).

[b21] Lara M. *et al.* Cold and ultracold dynamics of the barrierless D^+^ +H2 reaction: Quantum reactive calculations for ~R^−4^ long range interaction potentials. J. Chem. Phys. 143, 204305 (2015). 26627957 10.1063/1.4936144

[b22] Miklavc A., Markovic N., Nyman G., Harb V. & Nordholm S. Mechanism of quasiresonant vibration–rotation energy transfer in atom–diatom encounters. J. Chem. Phys. 97, 3348–3356 (1992).

[b23] Forrey R. C., Balakrishnan N., Dalgarno A., Haggerty M. R. & Heller E. Quasiresonant energy transfer in ultracold atom-diatom collisions. Phys. Rev. Lett. 82, 2657–2660 (1999).

[b24] Magill P. D., Stewart B., Smith N. & Pritchard D. E. Dynamics of Quasiresonant Vibration-Rotation Transfer in Atom-Diatom Scattering. Phys. Rev. Lett. 60, 1943–1946 (1988). 10038183 10.1103/PhysRevLett.60.1943

[b25] Rappoport D. & Furche F. Property-optimized Gaussian basis sets for molecular response calculation. J. Chem. Phys. 133, 134105 (2010). 20942521 10.1063/1.3484283

[b26] Kaupp M. *et al.* Pseudopotential approaches to Ca, Sr, an Ba hydrides. Why are some alkaline earth MX_2_ Compounds Bent? J. Chem. Phys. 94, 1360–1366 (1991).

[b27] Werner H.-J. & Knowles P. J. An efficient internally contracted multiconfiguration–reference configuration interaction method. J. Chem. Phys. 89, 5803 (1988).

[b28] Werner H.-J. & Knowles P. J. A second order multiconfiguration SCF procedure with optimum convergence. J. Chem. Phys. 82, 5053 (1985).

[b29] Buckingham A. D. Permanent and induced molecular moments and long-range intermolecular forces. Adv. Chem. Phys. 12, 107–142 (1967).

[b30] CRC Handbook of Chemistry and Physics 75 edn (ed Lide, D. R.) CRC Press (1995).

[b31] Chen K. *et al.* Molecular ion trap-depletion spectroscopy. Phys. Rev. A 83, 030501 (2011).

[b32] Goldflam R., Green S. & Kouri D. J. Infinite order sudden approximation for rotational energy transfer in gaseous mixtures. J. Chem. Phys. 67, 4149–4161 (1977).

[b33] Kouri D. J., Heil T. G. & Shimoni Y. Sufficiency conditions for the validity of the *j*_z_-conserving coupled states approximation. J. Chem. Phys. 65, 1462–1473 (1976).

[b34] McGuire P. Validity of the coupled states approximation for molecular collisions. Chem. Phys. 13, 81–94 (1976).

[b35] Stoecklin T., Voronin A. & Rayez J. C. Vibrational quenching of N_2_ (*v*=1,*J*_rot_=*j*) by ^3^He: Surface and Close-Coupling Calculations at Very Low Energy. Phys. Rev. A 66, 042703 (2002).

[b36] Ospelkaus S. *et al.* Quantum-state controlled chemical rections of ultracold potassium-rubidium molecules. Science 327, 853–857 (2010). 20150499 10.1126/science.1184121

[b37] O'Malley T. F., Spruch L. & Rosenberg L. Modification of Effective-Range Theory in the Presence of a Long-Range (r^-4^) Potential. J. Math. Phys 2, 491–498 (1961).

[b38] Joachain C. J. Quantum Collision Theory third edition North-Holland Publishing Company (1983).

